# The RNA-Dependent RNA Polymerase NIb of Potyviruses Plays Multifunctional, Contrasting Roles during Viral Infection

**DOI:** 10.3390/v12010077

**Published:** 2020-01-08

**Authors:** Wentao Shen, Yan Shi, Zhaoji Dai, Aiming Wang

**Affiliations:** 1 Key Laboratory of Biology and Genetic Resources of Tropical Crops, Ministry of Agriculture & Institute of Tropical Bioscience and Biotechnology, Chinese Academy of Tropical Agricultural Sciences, Haikou 571101, China; 2 College of Plant Protection, Henan Agricultural University, Zhengzhou 450002, China; shiyan@henau.edu.cn; 3 London Research and Development Centre, Agriculture and Agri-Food Canada, 1391 Sandford ST, London, ON N5V 4T3, Canada; zdai9@uwo.ca; 4 Department of Biology, University of Western Ontario, 1151 Richmond ST N., London, ON N6A 5B7, Canada

**Keywords:** *Potyvirus*, NIb, RNA-dependent RNA polymerase, virus-host interaction, autophagy, sumoylation, NPR1

## Abstract

Potyviruses represent the largest group of known plant RNA viruses and include many agriculturally important viruses, such as *Plum pox virus*, *Soybean mosaic virus*, *Turnip mosaic virus*, and *Potato virus Y*. Potyviruses adopt polyprotein processing as their genome expression strategy. Among the 11 known viral proteins, the nuclear inclusion protein b (NIb) is the RNA-dependent RNA polymerase responsible for viral genome replication. Beyond its principal role as an RNA replicase, NIb has been shown to play key roles in diverse virus–host interactions. NIb recruits several host proteins into the viral replication complexes (VRCs), which are essential for the formation of functional VRCs for virus multiplication, and interacts with the sumoylation pathway proteins to suppress NPR1-mediated immunity response. On the other hand, NIb serves as a target of selective autophagy as well as an elicitor of effector-triggered immunity, resulting in attenuated virus infection. These contrasting roles of NIb provide an excellent example of the complex co-evolutionary arms race between plant hosts and potyviruses. This review highlights the current knowledge about the multifunctional roles of NIb in potyvirus infection, and discusses future research directions.

## 1. Introduction

*Potyviridae* is the largest family of known plant RNA viruses and is comprised of 10 definitive and 3 proposed genera [1,2]. Among them, *Potyvirus* is the largest genus, including many agriculturally important viruses that cause serious diseases in many crops worldwide [1,2,3]. Example species in *Potyvirus* include *Potato virus Y* (PVY), *Plum pox virus* (PPV), *Soybean mosaic virus* (SMV), *Turnip mosaic virus* (TuMV), *Lettuce mosaic virus* (LMV), *Sweet potato feathery mottle virus* (SPFMV), and *Sugarcane mosaic virus* (SCMV), 4 of which have been ranked in the 10 most economically or scientifically important plant viruses [4,5]. Because of their importance as pathogens, potyviruses have been relatively more studied than other viruses [3]. In the past two decades, numerous studies have been directed to better understand the molecular plant–virus interactions for the development of novel antiviral strategies.

**Figure 1:**
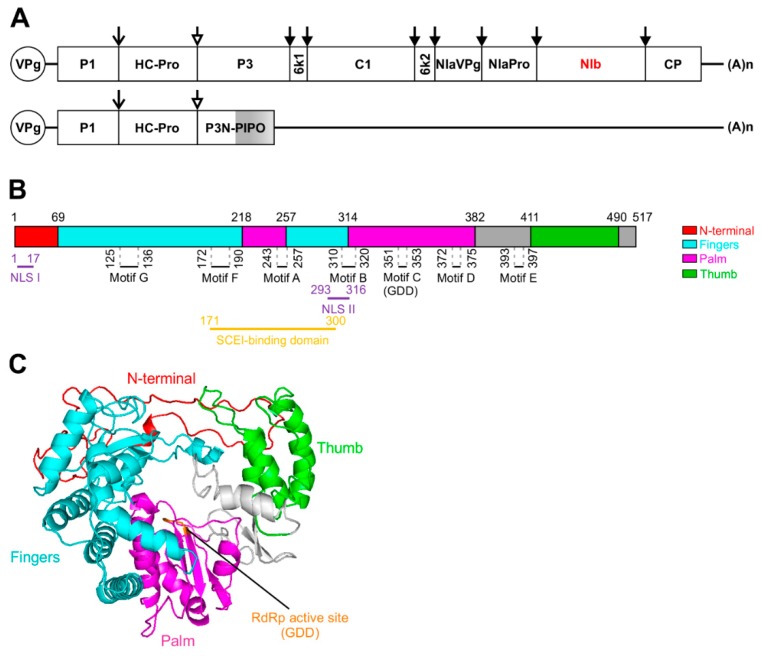
Schematic representation of the *Turnip mosaic virus* (TuMV) genome and the predicted structure of the TuMV nuclear inclusion b (NIb). (**A**) Schematic representation of the TuMV genome. The circle represents the genome-linked viral protein VPg, and (A)*n* represents the poly(A) tail. The open reading frame (ORF) is indicated as a long box. Mature proteins resulting from polyprotein processing are indicated by smaller boxes. PIPO derived from a frameshift on the P3 cistron is indicated as a grey box. P1 and HC-Pro release themselves by auto-proteolytic cleavage at their own C-termini. Other cleavages are processed by NIaPro. The mature proteins include coat protein (CP), nuclear inclusion b (NIb), which is the viral RNA-dependent RNA polymerase (RdRp), and two viral suppressors of RNA silencing (VSRs), HC-Pro and VPg. For clarity, the relative sizes of the mature proteins are not drawn to scale. (**B**) Schematic representation of TuMV NIb showing the predicted locations of RdRp signature motifs A to G. The locations of motifs A to G was determined by protein sequence alignment of TuMV NIb and RHDV RdRp. NLSs, the GDD motif, and the SCE1 binding domain are indicated. (**C**) Ribbon diagram of TuMV NIb predicted by the I-TASSER server. The TuMV NIb protein (NP_734221.1) sequence was submitted to the I-TASSER server for 3D structure prediction and the Rabbit hemorrhagic disease virus (RHDV) RdRp (PDB ID:1KHV) was identified as the most homologous protein in the PDB library. The typical RdRp fingers, palm, and thumb domains are colored cyan, magenta, and green, respectively, and the N-terminal domain is colored red. The RdRp active site is colored orange.

Potyviruses have a positive-sense, single-stranded RNA genome of approximately 10,000 nucleotides that encodes a long open reading frame (ORF) [1,2,3]. During virus replication, RNA polymerase slippage at the 5′ coding region leads to the generation of small sub-populations of viruses with shorter ORFs [1,2,3,6,7,8,9,10,11]. The large polyprotein is proteolytically processed by three viral proteases, i.e., P1 and helper component proteinase (HC-Pro) (each of which acts in *cis* on its own C-terminal end), and nuclear inclusion a protease (NIa-Pro), responsible for the remaining cleavage sites, to produce 10 functional proteins: P1, HC-Pro, P3, 6K1, cylindrical inclusion (CI) protein, 6K2, viral genome-linked protein (VPg), NIa-Pro, nuclear inclusion b (NIb), and capsid protein (CP) [1,2,3,6]. The small polyproteins consist of P3N-PIPO for most potyviruses, such as TuMV (Figure 1A), or other small transframe products such as P3N-ALT and P1N-PISPO for a few other potyviruses [7,8,9,10,11]. Potyviral NIb is the RNA-dependent RNA polymerase (RdRp) that is absolutely required for potyviral genome replication [12]. All RNA viruses including potyviruses replicate in the cytoplasm, and the process is catalyzed by the cytoplasmic membrane-bound viral replication complexes (VRCs) [13,14,15]. However, the NIb protein contains nuclear localization signals (NLSs) and has nuclear translocation activity. Consistently, it accumulates predominantly in the nucleus and together with NIa forms amorphous or crystalline nuclear inclusions (NIs) in infected cells [12,16,17,18]. Clustered point mutations within the NLSs of NIb that disrupt its nuclear translocation activity abolish viral genome replication, and this replication defection can be rescued in transgenic cells expressing a functional NIb [12]. Thus, NIb functions in *trans* and may need other viral-replication-associated proteins and/or host factors to deliver its RdRp activity in the VRC [19]. Like other potyviral proteins such as HC-Pro, CI, VPg, and CP, NIb is also a multifunctional protein [20,21,22,23,24,25].

## 2. Structural Characterization of NIb

The NIb coding sequence is located between the NIa-Pro cistron and the CP-coding sequence in the genomes of the majority of potyviruses (Figure 1A). The exceptions known so far include *Euphorbia ringspot virus* (the genus *Potyvirus*) and *Ugandan/cassava brown streak virus* (the genus *Ipomovirus*) in which the NIb is flanked by NIa-Pro and a Maf/HAM1 pyrophosphatase-like sequence [1]. During polyprotein processing, NIb is released by the NIa proteinase, which proteolytically processes peptides with conserved heptapeptide cleavage sites [26]. The minimal substrate length of cleavage site between NIb and CP of *Tobacco vein mottling virus* is four in the carboxy region and six in the amino region around the scissile bond [27]. In the case of PPV, the cleavage site between NIb and CP may be processed either in *cis* or in *trans* by the NIa protein but cleavage between NIa-Pro and NIb occurs in *cis* only [28,29]. *Tobacco etch virus* (TEV) also prefers cleavage in *trans* at the site between NIb and CP and in *cis* at the site between NIa-Pro and NIb [30]. It is possible that *cis* processing of the polyprotein at the site between NIa-Pro and NIb is forced by the polyprotein conformation in vivo. The potyviral NIbs have a molecular mass (MM) of nearly 60 kDa. The TuMV NIb contains 517 amino acids with an estimated MM of 59.6 kDa (Figure 1B).

It is well known that RdRp is the only universal viral protein encoded by all RNA viruses, making it an ideal natural target for evolutionary analysis of RNA viruses [31,32]. The viral RdRps are structurally arranged in a classical closed right-hand architecture formed by three subdomains named the palm, thumb, and fingers [33,34,35]. Most of the conservative structural elements of viral RdRps are contained in the palm subdomain, while the fingers and thumb subdomains are quite diverse among different RdRps. The palm subdomain contains five structural conservative motifs (A–E) [34,35]. The GDD sequence motif, which is essential for RdRp activity and is a hallmark of viral RdRps, is located in the C motif. The fingers subdomain includes two conserved motifs, F and G [36]. To the best of our knowledge, none of plant viral RdRps have been structurally characterized. Since plant potyviruses are closely related to animal picornaviruses [31,37], it is reasonable to suggest that the potyviral NIb may adopt similar regulatory mechanisms to the poliovirus RdRp (3Dpol) to deliver its core function. The 3Dpol, conserved among animal picornavirues, is also structurally arranged in a right-hand orientation, and contains seven conserved motifs (A–G)) [38]. Motifs A, B, D, and E are critical for the recognition and binding of nucleoside triphosphates; A and G for the binding of metal ions and the transfer of phosphoryl groups; D for the structural integrity of the palm; E for the binding of the priming nucleotide; and B, F, and G for the binding of the template [38,39,40]. Consistently, seven conserved motifs including SLKAEL (RNA polymerase activity), CVDDFN, CHADGS (RNA-dependent polymerase activity), GDD, [A/S]M[I/V]E[S/A]WG, FTAAP[L/I][D/E], and GNNSGQPSTVVDNTLMV (RNA polymerase activity) are present in the potyviral NIbs (Figure 1B) [41,42,43]. The NIb of the potyvirus TEV, the poliovirus 3Dpol (a picornavirus), and the RdRp of cowpea mosaic virus (CPMV, a comovirus)) share the highest sequence homology in two clusters, (T/S) GXXXTXXXN (T/S) and GDD [44]. Mutants with substitutions affecting the conserved GDD motif or the NIa/NIb cleavage site are lethal in tobacco protoplasts [45]. The relocation of the *NIb* gene to other intercistronic positions may result in nonviable viruses except when NIb is placed at the amino terminus of the polyprotein or between P1 and HC-Pro [46], suggesting the existence of various restrictions to the organization of the potyviral genome. The self-interaction of NIb has been observed at least for some potyviruses such as SMV and *Shallot yellow stripe virus* [47]. Additionally, NIb may interact with other potyviral proteins such as NIa-Pro and VPg [47,48]. The RdRp–RdRp self-interaction seems to be a common feature for positive-sense, single-stranded RNA viruses, including insect-, animal- and human-, and plant-infecting viruses [38,49,50,51,52,53]. The dimerization or oligomerization of RdRps may increase the stability of these enzymes and protect against degradation.

## 3. NIb is More Than an RdRp in the VRC

As mentioned above, the membrane-bound VRC is responsible for the replication of positive-stranded RNA viruses. The essential components of the VRC include the viral RdRp, replication-associated viral and host proteins, and viral RNA, as well as its associated double-stranded RNA, the replicative form RNA [15,54,55]. The virus-induced VRC-containing membranous vesicles not only provide the scaffold to anchor the VRC but also safeguard double-stranded RNA replication intermediates from RNAi-mediated degradation [15,56]. Many plant and animal viruses remodel the endoplasmic reticulum (ER) to form membranous vesicles which accommodate the VRC [57]. For potyvirus replication, it is the viral membrane protein 6K2 or the 6K2-VPg-Pro precursor that induces the formation of the VRC-containing vesicles at the ER exit sites in a COPI- and COPII-dependent manner [14,58,59]. Subsequently, the 6K2-induced vesicles traffic to the Golgi apparatus via COPII vesicles, and then target the periphery of chloroplasts through the actomyosin motility system, suggesting that potyviruses sequentially recruit the ER and chloroplasts for replication [15,60]. Recently, the availability of powerful three-dimensional (3D) imaging technologies such as electron tomography (ET) has made it possible to visualize the membrane structures induced by viruses at an unprecedented high resolution [61,62]. Indeed, an ET-based 3D reconstruction of images of ultrathin sections of TuMV-infected cells revealed that the single-membrane vesicle-like structures (SMVLs) and double-membrane vesicle-like structures (DMVLs) observed previously using regular transmission electron microscopy (TEM) are actual tubules [61]. Further immunoelectron microscopy localized dsRNA and viral-replication-required proteins to the single-membrane vesicle tubules (SMTs), suggesting that the SMTs are the true sites of TuMV replication [61].

In addition to NIb and 6K2, several viral proteins including HC-Pro, P3, CI, and NIa have been shown to be key components of the VRC [54,63,64]. Earlier studies suggested that NIb is likely recruited to the VRC via its interaction with the VPg domain of 6K2-VPg-Pro [12,65,66]. The recruitment of viral RNA to the VRC occurs via the interaction between NIb and the secondary structures at the 3′ untranslated region of the viral RNA [67,68]. Like the members of the family *Picornaviridae*, potyviruses exclusively employ the protein-primed mechanism of initiation of both minus and plus-strand RNA synthesis. The potyviral VPg is possibly uridylated by NIb by a similar mechanism to the 3Dpol of picornaviruses, and the resulting VPg-oligo-uridylate serves as a primer [69,70].

NIb is probably the most “sticky” among potyviral proteins, as it is a very active recruiter that interacts with many pro-viral host proteins and co-opts them to promote viral infection. Through the interactions with NIb, many of them are recruited to the VRC. A poly(A)-binding protein (PABP) of cucumber was the first host factor identified to interact with the NIb of a potyvirus (*Zucchini yellow mosaic virus*) [71]. Subsequent studies discovered that PABP2, PABP8, eukaryotic elongation factor 1A (eEF1A), heat shock cognate protein (Hsc or HSP) 70-3, and an RNA helicase-like protein in *Arabidopsis* (AtRH8) also interact with the potyviral NIb [72,73,74,75,76]. These NIb-interacting host proteins are present in the VRC and are important for viral genome multiplication, although the underlying molecular mechanisms remain to be determined experimentally. It is worth to mention that several of these host proteins are translation-related, suggesting their possible involvement in viral genome translation. Moreover, both eEF1A and Hsc70 are also important components of the VRC of some non-potyviruses [77,78]. Since TuMV translation and replication are tightly coupled within 6K2-induced vesicles [19,54], it has been suggested that some of them may participate in the regulation between potyviral RNA replication and translation [72,75,79]. Recently, we have shown that AtRH9, an RNA helicase from *A. thaliana* and an α-expansin (NbEXPA1) from *Nicotiana benthamiana* are two crucial host factors of TuMV [80,81]. The accumulation of TuMV is adversely affected in the *AtRH9* knockout or *NbEXPA1* knockdown plants. Since both AtRH9 and NbEXPA1 interact with NIb and are present in the VRC, it is possible that such interactions promote viral replication by stimulating NIb’s RdRp activity. In addition, AtRH9, as an RNA helicase, may participate in the separation of RNA duplexes during viral genome replication [80]. NbEXPA1 is a plasmodesmata (PD)-specific cell-wall-loosening protein. The cell-to-cell movement of plant viruses occurs through PD [82]. The NbEXPA1–NIb interaction reduces the accumulation level of NbEXPA1 at PD, which may compromise the canonical function of NbEXPA1 in plant growth and development, leading to activating antiviral defense pathways. Therefore, transient overexpression of NbEXPA1 promotes viral replication and cell-to-cell movement [81].

## 4. NIb and Sumoylation 

In virally infected cells, viral protein function may be modulated by post-translational modifications (PTMs), including phosphorylation, ubiquitination, methylation, and sumoylation [83,84,85]. Sumoylation refers to a highly dynamic, transient, reversible PTM process by which a small ubiquitin-like modifier (SUMO) family of proteins is covalently conjugated to lysine residue(s) of target proteins [86]. Sumoylation may affect the structure, subcellular localization, and enzymatic activity of the target proteins. In TuMV-infected cells, NIb is sumoylated by small ubiquitin-like modifier 3 (SUMO3) via SUMO-conjugating enzyme 1 (SCE1) [87,88]. The SCE1 binds to the central region of NIb to initiate sumoylation [88]. After sumoylation, the distribution of NIb shifts from the nucleus to the cytoplasm where viral replication takes place [87]. These findings suggest that sumoylation regulates the nuclear–cytoplasmic partitioning of NIb. On the other hand, SUMO3 plays a role as an antiviral defender by sumoylation of NONEXPRESSER OF PATHOGENESIS-RELATED GENES1 (NPR1) to activate the NPR1 resistance pathway [89]. NPR1 is a master regulator of the plant hormone salicylic acid (SA)-mediated plant immunity [90,91]. In the nucleus, NPR1 activates the expression of PR (PATHOGENESIS-RELATED) genes to promote defense responses [92]. These data suggest that after viral genome translation, NIb targets the nucleus, where it competes against NPR1 for and/or depletes SUMO3 to suppress TuMV-infection-induced, SUMO3-activated-NPR1-mediated immune response [87]. Therefore, in addition to its roles as an RNA polymerase of the VRC and a component recruiter for the VRC, NIb may function as a suppressor of host defense response to promote potyvirus infection.

## 5. NIb and Autophagy 

Autophagy is an evolutionarily conserved regulated mechanism of all eukaryotic cells that degrades and recycles defective organelles, toxic proteins and macromolecules, and various other aggregates to maintain cellular homeostasis during many biological processes such as development and abiotic and biotic stresses [93,94]. Accumulated evidence suggests that autophagy is involved in viral infection mainly as an antiviral mechanism by degradation of viral particles or viral proteins and/or effectors [95,96]. In a recent study, we showed that Beclin1 (also known as ATG6), a core component of autophagy, interacts specifically with TuMV NIb, and then targets NIb to autophagosomes for degradation [97]. The Beclin1-mediated NIb degradation requires a key autophagic adaptor protein, ATG8a, which interacts with Beclin1 to facilitate the docking of the Beclin1–NIb or Beclin1–NIb–VRC complex to autophagosomes. Interestingly, the overexpression of a truncated Beclin1 mutant that binds to NIb but is unable to mediate NIb degradation also suppresses viral replication, suggesting that Beclin1 can suppress NIb activity independent of Beclin1-mediated autophagy [96]. The interaction was mapped to the GDD motif of NIb. As the GDD motif is conversed among the RdRps of RNA viruses, it is not surprised that Beclin1 also interacts with and suppresses all RNA viruses examined, including three additional potyviruses (TEV, PPV, and SMV) and two nonpotyviruses (*Cucumber green mottle mosaic virus* of the genus *Tobamovirus*, and *Pepino mosaic virus* of the genus *Potexvirus*) [97]. 

Besides NIb, another important viral protein, HC-Pro, which is the major potyviral VSR, is also a target of autophagy. In this case, it has been shown that both host proteins rgs-CaM, a calmodulin-like protein, and NBR1, an autophagy cargo receptor, can mediate the degradation of HC-Pro via autophagy to restrict virus infection [96,98]. Moreover, like many animal viruses, potyviruses have evolved some strategies to subvert autophagy to promote their infection [99]. For instance, suppressor of gene silencing 3 (SGS3) and its intimate partner RNA-dependent RNA polymerase 6 (RDR6) are two crucial components of RNA silencing. The VPg protein (the second VSR of potyviruses) may interact with SGS3 and mediate the degradation of SGS3 and RDR6 via both ubiquitin and autophagy pathways [100].

## 6. NIb and Pathogenesis 

Among a number of antiviral defense mechanisms deployed by plants to restrict viral infection [101,102,103], the dominant gene (*R*)-conferred genetic resistance is among the most effective strategies to control viral pathogens for crop production. This type of resistance works based on a well-known gene-for-gene interaction model [104,105]. In brief, a plant-encoded *R* gene product recognizes a specific viral effector, known as avirulence factor (Avr), to activate effector-triggered immunity (ETI), which confers strong resistance and is often associated with the hypersensitive response (HR) [106,107]. R proteins mostly belong to the nucleotide-binding site–leucine-rich repeat (NB-LRR) family [108]. In pepper, a potyvirus resistance gene *Pvr4* from Criollo de Morelos 334 (CM334) variety confers dominant resistance to six potyviruses, including *Pepper mottle virus* (PepMoV), *Pepper severe mosaic virus* (PepSMV), and PVY [109]. The protein encoded by Pvr4 recognizes the NIbs of these potyviruses to trigger this resistance [110,111]. NIb is the avirulence factor for the *Pvr4* gene [111,112]. Pvr4 is a coiled-coil (CC) NB-LRR-type protein, and its LRR domain is a crucial region for the recognition of the avirulence effector NIb [112]. The CC domain is responsible for the induction of autoactive cell death in *N. benthamiana,* but its activity is suppressed by the nucleotide-binding-Apaf1-R protein-CED4 (NBARC) and LRR domain in the absence of NIb [112]. When Pvr4 recognizes NIb, the interaction between NIb and the LRR domain of Pvr4 releases the suppression of the CC domain, thereby activating the cell death response [112]. Therefore, the potyviral NIb functions as an ETI elicitor to inhibit viral infection. Consistent with this, the NIb of the PVY MsNr strain was found to be the elicitor of a veinal necrosis–HR response in a tobacco line carrying the *Rk* gene, which provides resistance to a root knot nematode [113]. 

*Pvr4* not only confers broad-spectrum resistance to potyviruses but also shows durability under field conditions [110,114]. So far, no PVY strains that can breach *Pvr4*-conferred resistance in the field have been documented. The evolutionary constraint acting on NIb is considered a major factor influencing the durability of *Pvr4* [110]. As NIb is one of the most constrained proteins among potyviruses, amino acid substitution in NIb is likely to induce a high fitness penalty in their natural primary hosts [114,115]. This assumption has been supported by the observation that a single nonsynonymous nucleotide substitution in the NIb cistron of PVY decreases the competitiveness and fitness of the virulent PVY mutant in susceptible pepper plants [110]. In contrast to this, a specific mutation occurring in the NIb of PPV was found to help the virus better adapt to pea, a new host in which the PPV accumulation level increased up to 20 times in comparison with that in peach, a natural host [116]. Thus, the mutation–fitness penalty assumption does not apply to the new hosts. The exact underlying mechanism has not been characterized. We speculate that the increased replication ability in the new hosts may result from the enhanced interaction of the mutated NIb with some host factors. In a recent study, Zhang et al. showed that the wheat yellow mosaic virus NIb interacts with the wheat light-induced protein TaLIP to facilitate viral infection by disruption of the ABA signaling pathway [117]. Taken together, these findings suggest a functional role of NIb as a pathogenesis determinant at least in some potyvirus–host combinations.

## 7. Conclusions and Future Prospects

**Figure 2:**
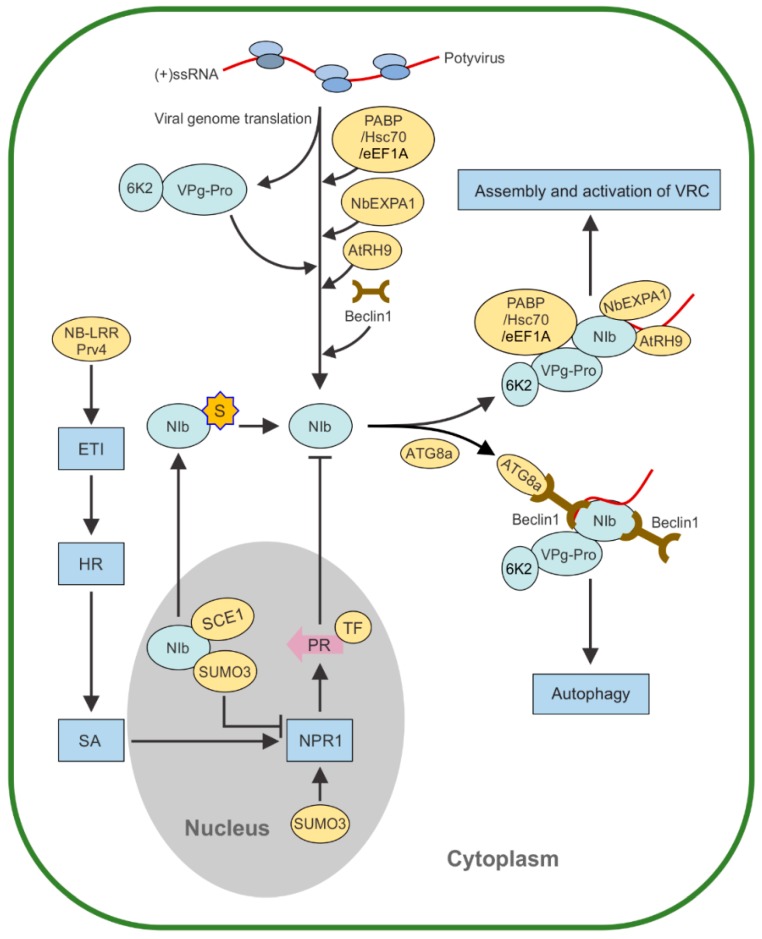
NIb plays contrasting roles in potyvirus infection. After entry into host plant cells, a potyvirus virion undergoes disassembly of viral particles and viral genome translation to produce viral proteins required for replication. The 6K2 or its precursor remodels the ER to form the VRC-containing vesicles at ER exit sites for potyvirus genome replication. The 6K2-induced vesicles may subsequently target chloroplasts for robust viral replication. NIb is recruited to the VRC, likely via its interaction with VPg domain of 6K2-VPg-Pro. NIb recruits many host factors such as poly(A)-binding protein (PABP), eukaryotic elongation factor 1A (eEF1A), heat shock cognate 70-3 (Hsc70-3), A. thaliana RNA helicase AtRH9, and N. benthamiana α-expansin (NbEXPA1). Potyvirus infection activates autophagy in plants and Beclin1 (ATG6) interacts with NIb to directly inhibit NIb activity or mediate autophagic degradation of NIb to restrict virus infection. Beclin1-mediated NIb degradation requires a key autophagic adaptor protein, ATG8a, which targets to Beclin1 to facilitate the docking of the Beclin1–NIb or Beclin1–NIb–VRC complex to autophagosomes. NIb may also act as an avirulence factor to activate ETI by recognition of resistance gene R-encoded proteins such as Pvr4. In addition, the nuclear-localized NIb is sumoylated by SUMO3. The sumoylation of NIb promotes potyvirus infection by relocalization of NIb from the nucleus to the cytoplasm and inhibition of the SUMO3-activated-NPR1-mediated resistance pathway.

In summary, potyviral NIb is a multifunctional protein, and functions as much more than just an RdRp in the viral infection process. It is a recruiter that co-opts proviral host proteins involved in the assembly and activation of the VRC, and a suppressor of host defense response that specifically tackles the NPR1-mediated immunity signaling pathway. Meanwhile, it is also a target of host antiviral defense, e.g., as a substrate of selective autophagy, and furthermore it is an elicitor that activates ETI. NIb’s multifunctionality is attributed to the complex interaction network of NIb, and establishes the significance of NIb in the co-evolutionary arms race between plants and potyviruses. We summarize the diverse functions of NIb in the potyviral infection in Figure 2.

Although extensive studies have been devoted to the characterization of NIb’s roles in potyvirus infection in the past 30 years, much more remains to be explored. To date, we still do not understand clearly how NIb joins other viral proteins and host factors to form the VRC. We still cannot establish an active in vitro viral replicase complex to study potyvirus replication. The availability of this cell-free system would significantly accelerate research on the potyvirus replication mechanism and characterization of the components essential for virus replication. Since RNA viral replication is always associated with cellular membranes [61,62], it would be interesting to conduct high-resolution analyses of the architecture of the membrane structures associated with the potyvirus VRC using advanced ET 3D technology, and investigate the possible role of NIb in remodeling membranous structures and anchoring the VRC to the membrane [62]. It has been suggested that potyviral genome translation and replication are a coupled process [20]. One further important question to be answered is how these two steps are balanced and coordinated to avoid collision, and how the VRC switches from biosynthesis of the negative-strand viral RNA to the plus-strand RNA. Crystallization and cryoelectron microscopy techniques could be used to study the ultramicrostructure of NIb or the NIb complex [23,118,119,120]. These techniques could also be employed to investigate spatial and temporal high-resolution structures of plant viral particles in virally replicating cells [121]. Such studies would certainly help us better understand the structure-based function of NIb and advance the knowledge of how the virus switches from replication to assembly and cell-to-cell movement. In this review, we briefly discussed the involvement of sumoylation in the nuclear-cytoplasmic partitioning of NIb [87]. However, how the SUMO3-sumoylated NIb is transported from the nucleus to the cytoplasm is yet to be understood. It remains unclear precisely how potyviruses regulate the distribution of the amounts of NIb in the nuclear and cytoplasm to maximize the benefit for the potyviral replication. The molecular identification of more host factors that interact with NIb, and understanding of mechanistic details of these new host factors as well as those known ones, will improve our understanding of the potyviral infection process and assist in the development of novel effective antiviral strategies for sustainable crop production.
